# *Pfkelch13 Plasmodium falciparum* Mutations in Huambo, Angola

**DOI:** 10.3390/pathogens11050554

**Published:** 2022-05-08

**Authors:** Ana Beatriz Batista Rodrigues, Rebecca de Abreu-Fernandes, Zoraima Neto, Domingos Jandondo, Natália Ketrin Almeida-de-Oliveira, Aline Rosa de Lavigne Mello, Joana Morais, Cláudio Tadeu Daniel-Ribeiro, Didier Menard, Maria de Fátima Ferreira-da-Cruz

**Affiliations:** 1 Laboratório de Pesquisa em Malária, Instituto Oswaldo Cruz, Fundação Oswaldo Cruz (Fiocruz), Rio de Janeiro 21040-900, Brazil; anabroodrigues@gmail.com (A.B.B.R.); rebeccasantos@aluno.fiocruz.br (R.d.A.-F.); nataliaketrin@gmail.com (N.K.A.-d.-O.); aline.lavigne@ioc.fiocruz.br (A.R.d.L.M.); ctdr@uol.com.br (C.T.D.-R.); 2 Centro de Pesquisa, Diagnóstico e Treinamento em Malária (CPD-Mal)/Reference Center for Malaria in the Extra-Amazonian Region of the Brazilian Ministry of Health, SVS & Fiocruz, Rio de Janeiro 21040-900, Brazil; 3 Instituto Nacional de Investigação em Saúde (INIS), Ministério da Saúde, Luanda 999104, Angola; zoraima.neto@gmail.com (Z.N.); domingos-jandondo@hotmail.com (D.J.); jmafonso.7@gmail.com (J.M.); 4 Institut Pasteur, INSERM U1201, 75015 Paris, France; didier.menard@pasteur.fr; 5 Institute of Parasitology and Tropical Diseases, UR7292, Dynamics of Host-Pathogen Interactions, Federation of Translational Medicine, University of Strasbourg, 67081 Strasbourg, France; 6 Laboratory of Parasitology and Medical Mycology, Strasbourg University Hospital, 67081 Strasbourg, France

**Keywords:** malaria, *Plasmodium falciparum*, *pfk13*, artemisinin, ACTs, Angola, resistance

## Abstract

Artemisinin (ART) is recommended as the first-line drug for *P. falciparum* infections combined with a long-acting partner drug. The emergence of *P. falciparum* resistance to ART (ARTR) is a concern for malaria. The most feared threat remains the spread of ARTR from Southeast Asia to Africa or the independent emergence of ARTR in Africa, where malaria accounts for 93% of all malaria cases and 94% of deaths. To avoid this worst-case scenario, surveillance of *Pfkelch13* mutations is essential. We investigated mutations of *Pfkelch13* in 78 *P. falciparum* samples from Huambo, Angola. Most of the parasites had a wild-type *Pfkelch13* allele. We identified one synonymous mutation (R471**R**) in 10 isolates and one non-synonymous mutation (A578**S**) in two samples. No *Pfkelch13* validated or candidate ARTR mutants were identified. The finding suggests that there is little polymorphism in *Pfkelch13* in Huambo. Since cases of late response to ART in Africa and the emergence of ARTR mutations in Rwanda and Uganda have been reported, efforts should be made toward continuous molecular surveillance of ARTR. Our study has some limitations. Since we analyzed *P. falciparum* parasites from a single health facility, the study may not be representative of all Angolan endemic areas.

## 1. Introduction

Malaria is an endemic disease of mandatory notification, caused by *Plasmodium* parasites that are transmitted to humans by infected female *Anopheles* mosquito bites. It is estimated that among the 100 species of *Plasmodium* only eight of them are able to infect humans: *P. falciparum*, *P. vivax*, *P. malariae*, *P. ovale curtisi*, *P. ovale wallikeri*, *P. simium* [1], *P. knowlesi* [2], and *P. cynomolgi* [3].

This endemic disease remains a global public health problem threatening the most vulnerable populations in Africa, South and Central America, and Asia. Malaria attacks over 241 million people, with at least 627,000 deaths annually, with 77% of those deaths occurring in children under the age of five, and 94% of them occurring in Africa [4]. Notably, in Africa, these malaria figures are worsening due to the SARS-COVID 19 pandemic, which has compromised malaria treatment and control measures [5].

*P. falciparum* is the species responsible for the highest death rate, mainly in sub-Saharan Africa, where the disease has never been controlled [6].

Besides controlling the vector—an important measure for controlling the spread of the disease—prompt diagnosis and treatment must be implemented in endemic countries to cure patients, eliminate the sexual and asexual blood stages, and prevent the transmission of parasites to the mosquito vector [7]. The control of the disease also depends on the availability and adequate use of effective antimalarial drugs; therefore, antimalarial drug resistance is a serious obstacle [8]. *P. falciparum*’s resistance to previous generations of drugs, such as chloroquine and sulfadoxine-pyrimethamine (SP), became widespread in the 1950s and 1960s. Since then, resistance to other alternative drugs has been increasingly reported. Currently, artemisinin (ART) is the most effective drug available for malaria therapy. Meanwhile, in 2007, in Cambodia, cases of ART-resistant *P. falciparum* parasites were reported, and since then, this resistance has continued spreading across Southeast Asia [9,10]. Consequently, in 2013, an emergency proposal related to artemisinin resistance (ARTR) was launched by the WHO, whose main objective was to prevent the spread of resistant parasites, mainly to African countries, where 93% of cases of falciparum malaria occur.

In this scenario, the implementation of immediate actions aimed at the elimination of malaria by 2030, includes priority measures focused on monitoring the appearance of *P. falciparum* ARTR parasite populations in endemic areas of Africa, South and Central America, and the Caribbean, besides interventions targeting the Mekong areas [4]. Mutations in the *Pfkelch13* helix domain, notably that identified on the Asian continent as C580**Y**, are associated with ARTR *in vitro* and *in vivo* [10,11]. The *Pfkelch13* gene encodes a 726-amino acid protein that consists of a specific N-terminal region of the Apicomplexa with three somewhat conserved domains: one comprising codons 212–341, the other comprising codons 350–437, and another domain that corresponds to a Kelch-repeat C-terminal helix comprising codons 443–726, which harbors the vast majority of *Pfkelch13* polymorphisms associated with ARTR [12].

Considering that molecular surveillance of ART resistance-associated mutations is essential for maintaining the useful life of this drug and the gains in child survival in Africa, here, we investigated mutations in the *Pfkelch13* gene of *P. falciparum* parasites from Huambo, a state of Angola.

## 2. Results

**Figure 1:**
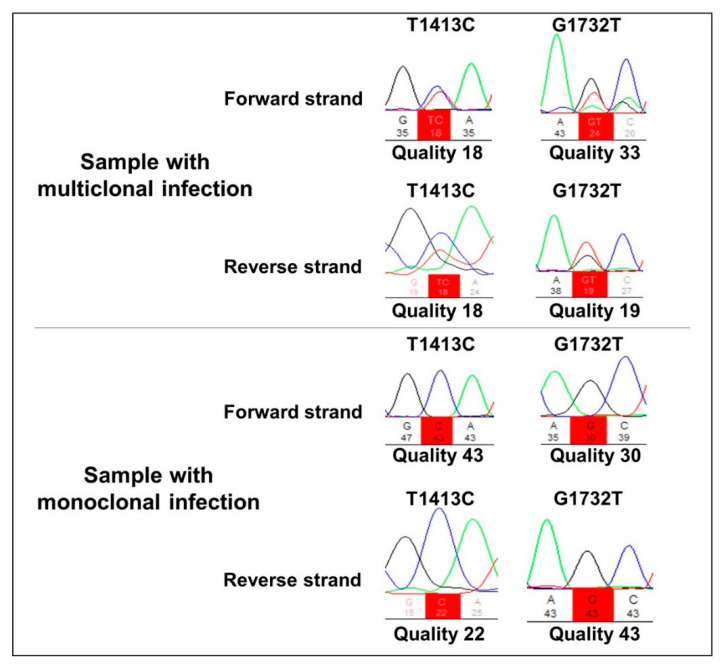
Representative electropherogram of multi-clone and monoclonal infections in the T1413**C** and G1732**T** nucleotide positions, corresponding to the amino acids R471**R** and A578**S**, respectively.

All 78 *P. falciparum* samples were amplified using *Pfkelch13* primers. The R471**R** synonym SNP was detected in 10 samples (13%), whereas the non-synonymous A578**S** polymorphism was found in two samples (2.5%). The two samples containing A578**S** also presented the R471**R** polymorphism, showing a multiclonal infection (Figure 1). All falciparum malaria patients were aparasitemic on Day 3 according to their thick blood smears, indicating the absence of parasites with the slow clearance phenotype, a measure of ARTR *in vivo* [13].

Among the *P. falciparum* samples, 66 (85%) presented a haplotypic profile identical to the 3D7 reference strain (FGNLCRTMAYVGATVPGNRIPVERMVPRAMCDEEQSIA), which was denominated the T0 haplotype. Two other haplotypes, named T1 and T2, were also identified: T1 (R471**R)** was detected in 10 (13%) samples, and T2, which comprises both R471**R** + A578**S** SNPs, was detected in two (2%) samples (Table 1).

## 3. Discussion

In Angola, artemisinin combined treatments (ACT) have been used nationwide since 2007 [14]. Thus, the samples here analyzed were collected 10 years after the introduction of ACT in the Angola National Treatment guidelines. Only two *Pfkelch13* mutants were detected: the non-synonymous (A578**S**) and the synonymous (R471**R**) mutations. The R471**R** mutant had been previously detected in the Democratic Republic of Congo (DRC) [15] and Gabon [16], as well as in Angola in two (4%) of the *P. falciparum* samples, which were collected in Malanje (one) and Luanda (one) in 2010, although prior to the introduction of ACT in 2003, this mutation was not reported [14]. Here, 13% of the *P. falciparum* samples from Huambo collected in 2017 presented the R471**R** (synonymous) *pfk13* polymorphism, showing an increase in this SNP compared with 2010 in Angola. Even though a synonymous mutation is not reflected in the protein sequence, it seems that the shift in drug policy in Africa towards ACTs has been accompanied by selection for k13 polymorphisms. In fact, three non-synonymous (A578**S,** M579**I**, and Q613**E**) and two synonymous (R471**R** and R575**R)** mutations were found in Angola, showing a total mutation rate of 3.8% from 2012 to 2017 [17].

We also observed the non-synonymous polymorphism A578**S**, a *Pfkelch13* mutant frequently reported in *P. falciparum* samples from Sub-Saharan African countries (Comoros Islands, Kenya, Uganda [18,19], Ghana, Congo, DRC, Gabon, Kenya, Mali, and Rwanda [20,21]) as well as in samples from Asia (southern Bangladesh and Cambodia [18]). These findings reflect that this mutation in the *Pfkelch13* helix of *P. falciparum* parasites has a global distribution and is a common polymorphism across Africa since this mutation was not related to *in vivo* and *in vitro* ARTR [22]. It remains to elucidate whether the presence of the A578**S** mutation in Huambo is the result of an independent appearance or if this variant came from other Angolan endemic areas. Indeed, the migration of people from the south (Huambo) to the north (Luanda, Malanje) searching for better living conditions (e.g., employment, education, goods, animals, and food transport) was very common after the civil war ended.

In response to antimalarial drug pressure, non-synonymous polymorphisms may be selected in the *Pfkelch13* helix region to favor parasite survival. The survival of the mutant parasites would therefore be the result of adapting to an oxidative stress environment, such as that generated by ART. This selective advantage would be reflected, for example, in an increase in the time to eliminate parasitemia. C580**Y** and M579**I** incur substantial fitness costs, which may slow their dissemination in high-transmission settings, in contrast with R561**H**, which, in African 3D7 parasites, is fitness-neutral [23].

To date, wild-type K13 continues to be dominant and few cases of late response to ART treatment have been reported in Africa. However, the emergence of the artemisinin resistance-related K13 R561H mutation in Rwanda [20,21], and the A675V and C469Y mutations in Uganda were recently reported [24]; consequently, the potential for the emergence of ARTR *P. falciparum* parasites in the African continent cannot be disregarded. In this light, efforts should be made toward continuous molecular surveillance to detect early signs of ARTR to prolong the lifespan of ACT in Africa, including mutations outside the propeller domain such as P413A. This mutation was very recently identified in a West African strain from Mali exposed to 18 cycles of sequential artemisinin pressure [25].

## 4. Materials and Methods

### 4.1. Study Population and Sampling

**Figure 2:**
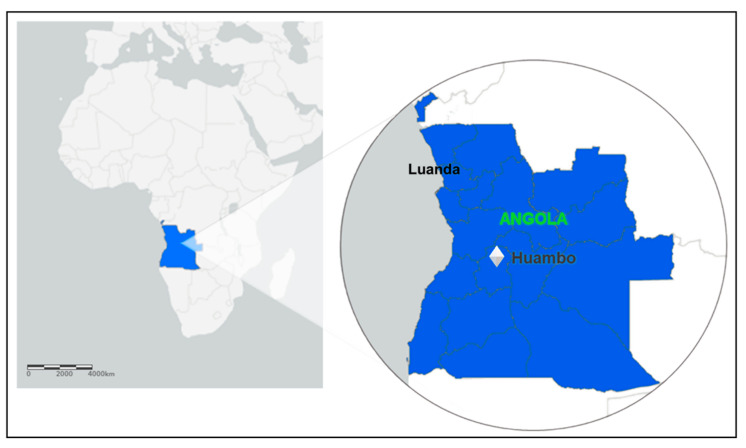
Map of Africa, highlighting the Luanda and Huambo provinces (states); ArcGIS free online interactive maps https://www.arcgis.com/home/webmap/viewer.html accessed on 4 March 2022.

In Angola, the entire country is endemic to malaria. There is heterogeneity in malaria transmission, ranging from low, seasonal, and epidemic-prone transmission in the dry south—which is the case for Huambo—to high, year-round transmission in the wet, tropical north of the country [26]. All patients engaged in the study are living in the municipality of Huambo, the capital of the province (state) of the same name. Huambo is a low-risk malaria area and corresponds to only 1% of Angolan malaria cases. The province of Huambo is moving toward the pre-elimination phase, a fact that motivated this study. According to the population projections of 2018 prepared by the National Statistics Institute (INE), Huambo has a population of 815,685 inhabitants and a territorial area of 2609 km², being the most populous municipality in the province and the seventh most populous in the country. After having a large part of its infrastructure destroyed by the war, it rebuilt economically after peace came in 2002. Located in the Central Plateau of Angola, the municipality has an altitude above 1774 m. The climate is characterized by humid and warm summers, with mild nights and relatively hot days, and dry winters with mild days and relatively cold nights. The city of Huambo is 513 km in a straight-line distance from Luanda, the capital of Angola (Figure 2).

Paper-dotted blood samples from febrile outpatients with axillary temperature ≥37.5 °C were collected in 2017 at the Huambo Central Hospital. In total, 78 blood spots were dried overnight, placed in individual bags with a desiccant, and then transported to the Fundação Oswaldo Cruz (Fiocruz) Malaria Research Laboratory in Rio de Janeiro, Brazil. In Fiocruz, DNA was extracted using QIAamp DNA mini kits (Qiagen, Hilden, Germany).

### 4.2. Ethical Aspects

The project was approved by the Ministry of Health of Angola (MINSA) Research Ethics Committee, in the context of a collaboration between the National Institute of Health Research (INIS, MINSA) and the Laboratório de Pesquisa em Malária, Fiocruz. Informed consent was obtained from all subjects involved in the study.

### 4.3. Malaria Diagnosis

The diagnosis of *P. falciparum* was performed by nested-PCR [27].

### 4.4. DNA Extraction, Amplification, and Sequencing

DNA extraction was performed using the QIAamp DNA blood mini kit (Qiagen, Hilden, Germany), according to the manufacturer’s instructions. Amplification of the *Pfkelch13* gene was conducted as previously described [9,28], using the following primers: K13 F: 5’ GGG AAT CTG GTG GTA ACA GC 3’ and K13 A: 3’ CGG AGT GAC CAA ATC TGG GA 5’ for the outer PCR, and K13 N_ F: 5’ GCC TTG TTG AAA GAA GCA GA 3’ and K13 N_R: 3’ GCC AAG CTG CCA TTC ATT TG 5’ for the inner PCR. Amplicons were purified using Wizard SV Gel and a PCR Clean-Up System kit (Promega, Wisconsin, EUA), according to the manufacturer’s instructions.

The sequencing reactions were performed with the same inner PCR primers (3.2 pmol/μL), according to the Big Dye Terminator Cycle Sequencing Ready Reaction version 3.1. protocol (Applied Biosystems, Waltham, MA, USA). DNA Sanger sequencing was performed on the ABI PRISM DNA Analyzer 3730 (Applied Biosystems, Waltham, MA, USA) of the PDTIS/Fiocruz genomic platform. The polymorphisms from 427 to 709 codons were examined.

### 4.5. Sequence Analyses of Polymorphisms 

Nucleotide sequences were aligned with the *P. falciparum* reference genome 3D7 strain (PF3D7_1343700) using the ClustalW multiple sequence aligner in BioEdit software [29]. The electropherogram was set to a cutoff score of 10 with NovoSNP10 [30] and Chromas version 2.6 software. DNA sequences were deposited in GenBank (the NIH’s genetic sequence database; www.ncbi/nlm/nih.gov/GenBank accessed on 4 May 2022) with the accession numbers OL456446–OL456519.

## Figures and Tables

**Table 1:** Haplotypes of *pfk13* in 78 *P. falciparum* samples from Huambo, Angola.

Haplotypes	DNA Target Sequence	Frequency
T0 ^1^	FGNLCRTMAYVGATVPGNRIPVERMVPRAMCDEEQSIA	66 (85%)
T1 ^2^	FGNLC**R**TMAYVGATVPGNRIPVERMVPRAMCDEEQSIA	10 (13%)
T2 ^3^	FGNLC**R**TMAYVGATVPGNRIPVERMVPR**S**MCDEEQSIA	2 (2%)

^1^ Reference Pf3D7 haplotype sequence. ^2^ The bold underlined character represents a synonymous mutation, exemplifying a single mutant haplotype. ^3^ The bold underlined character represents a synonymous mutation and the character in bold shows a non-synonymous mutation, demonstrating a double mutant haplotype in one sample.

## Data Availability

All data have been made available within the body of this article.
